# Individual-level factors associated with the risk of acquiring human *Plasmodium knowlesi* malaria in Malaysia: a case-control study

**DOI:** 10.1016/S2542-5196(17)30031-1

**Published:** 2017-06-09

**Authors:** Matthew J Grigg, Jonathan Cox, Timothy William, Jenarun Jelip, Kimberly M Fornace, Patrick M Brock, Lorenz von Seidlein, Bridget E Barber, Nicholas M Anstey, Tsin W Yeo, Christopher J Drakeley

**Affiliations:** Global and Tropical Health Division, Menzies School of Health Research and Charles Darwin University, Darwin, NT, Australia; Infectious Diseases Society Sabah-Menzies School of Health Research Clinical Research Unit, Kota Kinabalu, Sabah, Malaysia; London School of Hygiene & Tropical Medicine, London, UK; Infectious Diseases Society Sabah-Menzies School of Health Research Clinical Research Unit, Kota Kinabalu, Sabah, Malaysia; Jesselton Medical Centre, Kota Kinabalu, Sabah, Malaysia; Clinical Research Centre, Queen Elizabeth Hospital, Kota Kinabalu, Sabah, Malaysia; Sabah Department of Health, Kota Kinabalu, Sabah, Malaysia; London School of Hygiene & Tropical Medicine, London, UK; Institute of Biodiversity, Animal Health and Comparative Medicine, College of Medical, Veterinary and Life Sciences, University of Glasgow, UK; Mahidol-Oxford Research Unit, Bangkok, Thailand; Global and Tropical Health Division, Menzies School of Health Research and Charles Darwin University, Darwin, NT, Australia; Infectious Diseases Society Sabah-Menzies School of Health Research Clinical Research Unit, Kota Kinabalu, Sabah, Malaysia; Global and Tropical Health Division, Menzies School of Health Research and Charles Darwin University, Darwin, NT, Australia; Infectious Diseases Society Sabah-Menzies School of Health Research Clinical Research Unit, Kota Kinabalu, Sabah, Malaysia; Global and Tropical Health Division, Menzies School of Health Research and Charles Darwin University, Darwin, NT, Australia; Infectious Diseases Society Sabah-Menzies School of Health Research Clinical Research Unit, Kota Kinabalu, Sabah, Malaysia; Lee Kong Chian School of Medicine, Nanyang Technological University, Singapore; Communicable Disease Centre, Institute of Infectious Diseases and Epidemiology, Tan Tock Seng Hospital, Singapore; London School of Hygiene & Tropical Medicine, London, UK

## Abstract

**Background:**

The emergence of human malaria due to the monkey parasite *Plasmodium knowlesi* threatens elimination efforts in southeast Asia. Changes in land use are thought to be driving the rise in reported *P knowlesi* cases, but the role of individual-level factors is unclear. To address this knowledge gap we assessed human and environmental factors associated with zoonotic knowlesi malaria risk.

**Methods:**

We did this population-based case-control study over a 2 year period in the state of Sabah in Malaysia. We enrolled cases with microscopy-positive, PCR-confirmed malaria who presented to two primary referral hospitals serving the adjacent districts of Kudat and Kota Marudu. We randomly selected three malaria-negative community controls per case, who were matched by village within 2 weeks of case detection. We obtained questionnaire data on demographics, behaviour, and residential malaria risk factors, and we also assessed glucose-6-phosphate dehydrogenase (G6PD) enzyme activity. We used conditional logistic regression models to evaluate exposure risk between *P knowlesi* cases and controls, and between *P knowlesi* and human-only *Plasmodium* spp malaria cases.

**Findings:**

From Dec 5, 2012, to Jan 30, 2015, we screened 414 patients and subsequently enrolled 229 cases with *P knowlesi* malaria mono-infection and 91 cases with other *Plasmodium* spp infection. We enrolled 953 matched controls, including 683 matched to *P knowlesi* cases and 270 matched to non-*P knowlesi* cases. Age 15 years or older (adjusted odds ratio [aOR] 4·16, 95% CI 2·09–8·29, p<0·0001), male gender (4·20, 2·54–6·97, p<0·0001), plantation work (3·50, CI, 1·34–9·15, p=0·011), sleeping outside (3·61, 1·48–8·85, p=0·0049), travel (2·48, 1·45–4·23, p=0·0010), being aware of the presence of monkeys in the past 4 weeks (3·35, 1·91–5·88, p<0·0001), and having open eaves or gaps in walls (2·18, 1·33–3·59, p=0·0021) were independently associated with increased risk of symptomatic *P knowlesi* infection. Farming occupation (aOR 1·89, 95% CI 1·07–3·35, p=0·028), clearing vegetation (1·89, 1·11–3·22, p=0·020), and having long grass around the house (2·08, 1·25–3·46, p=0·0048) increased risk for *P knowlesi* infection but not other *Plasmodium* spp infection. G6PD deficiency seemed to be protective against *P knowlesi* (aOR 0·20, 95% CI 0·04–0·96, p=0·045), as did residual insecticide spraying of household walls (0·52, 0·31–0·87, p=0·014), with the presence of young sparse forest (0·35, 0·20–0·63, p=00040) and rice paddy around the house (0·16, 0·03–0·78, 0·023) also associated with decreased risk.

**Interpretation:**

Adult men working in agricultural areas were at highest risk of knowlesi malaria, although peri-domestic transmission also occurrs. Human behavioural factors associated with *P knowlesi* transmission could be targeted in future public health interventions.

**Funding:**

United Kingdom Medical Research Council, Natural Environment Research Council, Economic and Social Research Council, and Biotechnology and Biosciences Research Council.

## Introduction

*Plasmodium knowlesi* has emerged as a widespread cause of zoonotic human malaria in southeast Asia.^1^–^3^ The first naturally acquired human infection was described in 1965 in Peninsular Malaysia,^4^ with spill-over infections from the traditional monkey-vector transmission cycle presumed to be rare.^4^,^5^ However, modern molecular techniques assisted in the identification of a large focus of human *P knowlesi* symptomatic infections in Sarawak in Malaysian Borneo in 2004.^6^ *P knowlesi* cases have now been reported from all countries in southeast Asia, with the exception of Laos,^2^ that encompass the geographical ranges of the natural macaque hosts (*Macaca fascicularis* and *Macaca nemestrina*) and *Anopheles leucosphyrus* group vectors.^3^ In routine microscopy, *P knowlesi* is indistinguishable from *Plasmodium malariae,*^2^ but can also be misdiagnosed in knowlesi-endemic areas as *Plasmodium falciparum* and *Plasmodium vivax*, suggesting that its true incidence continues to be underestimated.^2^,^7^

Most notably, Malaysia has reported marked increases in knowlesi malaria incidence since 2004—a trend that cannot solely be explained by improved molecular *Plasmodium* species confirmation.^8^,^9^ Malaysia’s national malaria eradication programme has successfully reduced *P falciparum* and *P vivax* case numbers to a point where elimination of these species by 2020 is considered realistic.^10^ However, the control of zoonotic malaria transmission is likely to prove less tractable. *P knowlesi* is now the most common cause of malaria in Malaysia^11^ and is associated with the highest risk of severe disease.^11^,^12^

The drivers of the emergence of *P knowlesi* in endemic areas are thought to be predominantly related to changes in human land use,^13^,^14^ although the prevailing paradigm of risk being mostly limited to adult men with a history of forest exposure has been challenged by reports of peridomestic transmission, including within family clusters.^15^ Within this changing transmission context, more detailed evidence about determinants of risk is required to design and target appropriate interventions; however, to date, no formal studies have attempted to identify specific individual-level risk factors. To address this knowledge gap we did a case-control study to assess human and environmental factors associated with zoonotic knowlesi malaria risk.

## Methods

### Study design and participants

**Figure 1:**
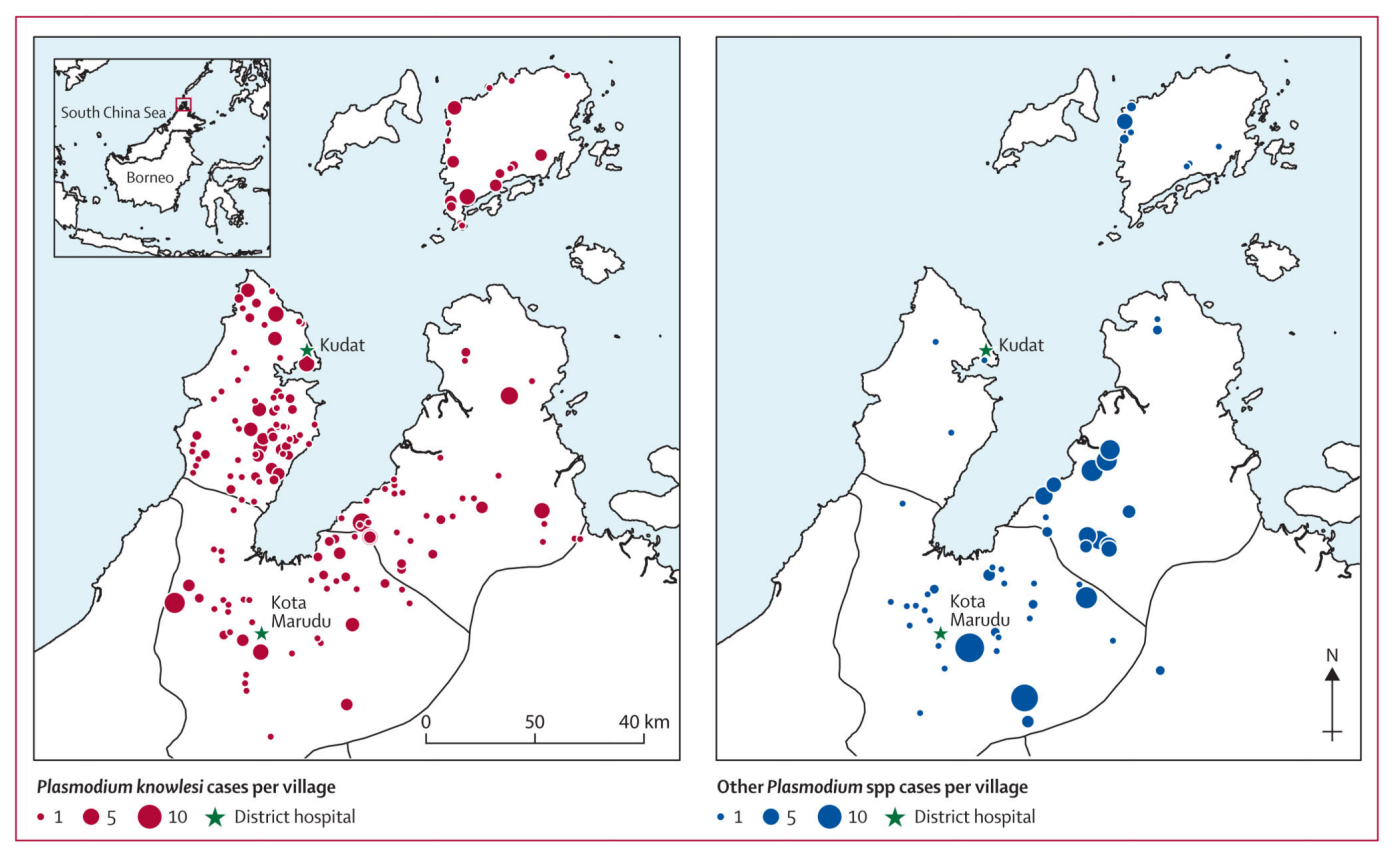
District study areas and malaria case distribution

In this population-based case-control study we examined factors associated with the risk of acquiring zoonotic knowlesi malaria in adults and children presenting to two primary referral hospitals serving the adjacent districts of Kudat and Kota Marudu in Sabah, east Malaysia (figure 1). The study design has been described previously.^16^ The study protocol was approved by the relevant human research ethics committees of Malaysia, the Menzies School of Health Research (Australia), and the London School of Hygiene & Tropical Medicine (UK).

The government referral hospitals provide free admission and malaria treatment and are accessible from all villages in the catchment area. National public health guidelines mandate microscopic malaria screening for all patients presenting to government facilities with fever and notification of all positive cases, thus increasing the likelihood of enrolling all symptomatic knowlesi malaria cases seeking care.

In brief, patients positive for *Plasmodium* species infection by microscopy were enrolled if they had a documented fever or history of fever in the past 48 h, had resided in the study catchment area in the previous 3 weeks, and had not been enrolled previously. We randomly selected controls from the case’s village within 2 weeks of case detection using updated Malaysian government village and household population data, and corroboration from health workers and village heads. To minimise selection bias, absent controls were revisited up to three times before an alternative household or individual was selected. We repeated the same selection process until three controls had been enrolled for each case. Inclusion criteria for controls were a negative blood slide for malaria, no history of fever in the preceding 48 h, not previously having been a case, not previously having been a control nor living in the same household as a control, and living at least 200 m away from the case household. We obtained informed consent for both cases and controls.

The selection criteria for controls were designed to allow the evaluation of micro-epidemiological human factors while minimising confounding effects from village-level forest cover, elevation, rainfall, and seasonality.^8^,^9^,^13^ Controls were not matched on age or gender in order to ascertain whether these factors might independently affect acquisition of symptomatic infection.

### Procedures

Cases were classified according to PCR-confirmed *Plasmodium* spp infection.^17^,^18^ Cases and controls completed identical pre-tested questionnaires and household surveys incorporating demographic, social, behavioural, household, and environmental variables associated with malaria acquisition risk, which were developed through prior expert and local consultation (appendix). Responses were restricted to the previous month to minimise recall bias. Household locations were established with a hand-held global positioning system (Garmin 62S, Garmin, Olathe, KS, USA). Blood sampling was done to measure haemoglobin (Hemocue, Angleholm, Sweden) and glucose-6-phosphate dehydrogenase (G6PD) enzyme activity (Beutler fluorescent spot test), as well as PCR for final *Plasmodium* species case confirmation. All female cases of childbearing age underwent pregnancy testing and pregnancy status for controls was self-reported. We entered data into electronic case record forms using Pendragon Forms VI software.

### Statistical analysis

With three controls per case, assuming a probability of exposure among controls of 0·1, and the relative risk of acquiring knowlesi malaria in exposed subjects relative to unexposed subjects of 0·3, a minimum of 210 *P knowlesi* cases were required to reject the null hypothesis that there is no difference between cases and controls (ie, relative risk of 1), with 80% power and an α of 0·05.^19^

The primary analysis of categorical exposure variables compared knowlesi malaria cases and controls as per protocol. We compared odds ratios (OR) and 95% CIs for exposure risk, calculated by the Mantel-Haenszel method. We included variables with a p value less than 0·2 on univariate analysis in the multivariate analysis within associated subgroups using conditional logistic regression models, including adjustment for age and gender. Variables with p values less than 0·2 then proceeded into the final multivariate model with adjusted odds ratios (aOR), followed by further reduction of predictor variables through a process of model-fitting using Bayesian information criterion (BIC) and backward step-wise exclusion of variables with p values greater than 0·05.

The secondary outcome compared knowlesi malaria cases with cases from other *Plasmodium* species combined (zoonotic *vs* non-zoonotic transmission). We assessed non-*P knowlesi* cases with matched controls with a similar approach to the primary univariate analysis, then made comparisons between *P knowlesi* and non-*P knowlesi* models for each exposure variable using Wald’s test.

Data were analysed with Stata 12.

## Results

**Figure 2:**
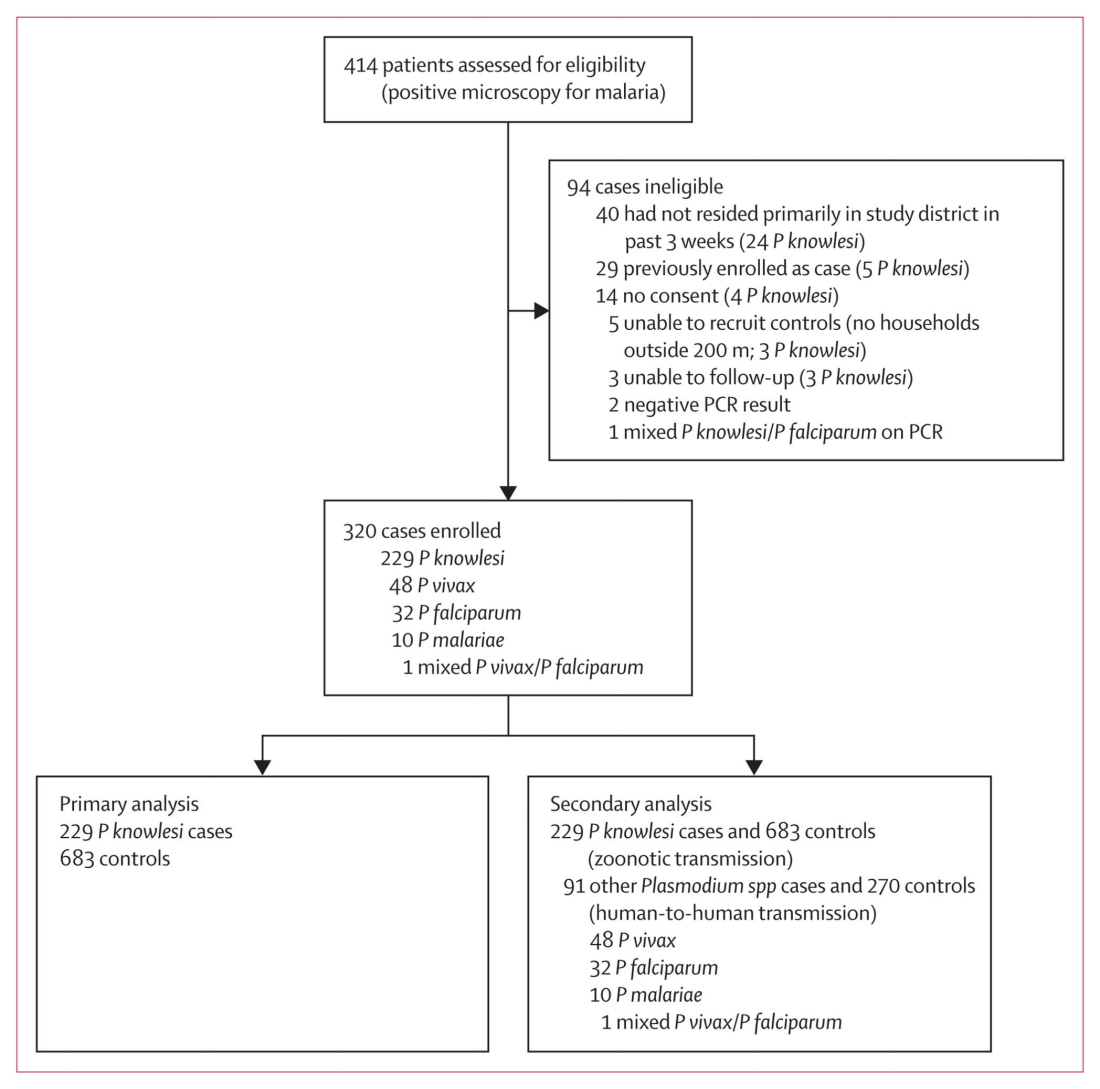
Study design *P knowlesi*=*Plasmodium knowlesi*. *P vivax*=*Plasmodium vivax*. *P falciparum*=*Plasmodium falciparum*. *P malariae*=*Plasmodium malariae*.

From Dec 5, 2012, to Jan 30, 2015, 414 patients with microscopy-positive malaria were screened for eligibility at the district sites (figure 2). We excluded a single remote area in eastern Kota Marudu district because of logistical difficulties with study procedures. Of these patients, 320 cases were enrolled, including 229 patients with symptomatic *P knowlesi* mono-infection and 91 with other *Plasmodium* species infection. 953 controls were enrolled, including 683 people matched to *P knowlesi* cases and 270 matched to non-*P knowlesi* cases. The table shows baseline features of cases and controls. Four *P knowlesi* cases had only two matched controls, due to the low number of households in these villages. One non-*P knowlesi* case had only one control enrolled and another non-*P knowlesi* case only had two controls enrolled because there were few households nearby.

**Figure 3:**
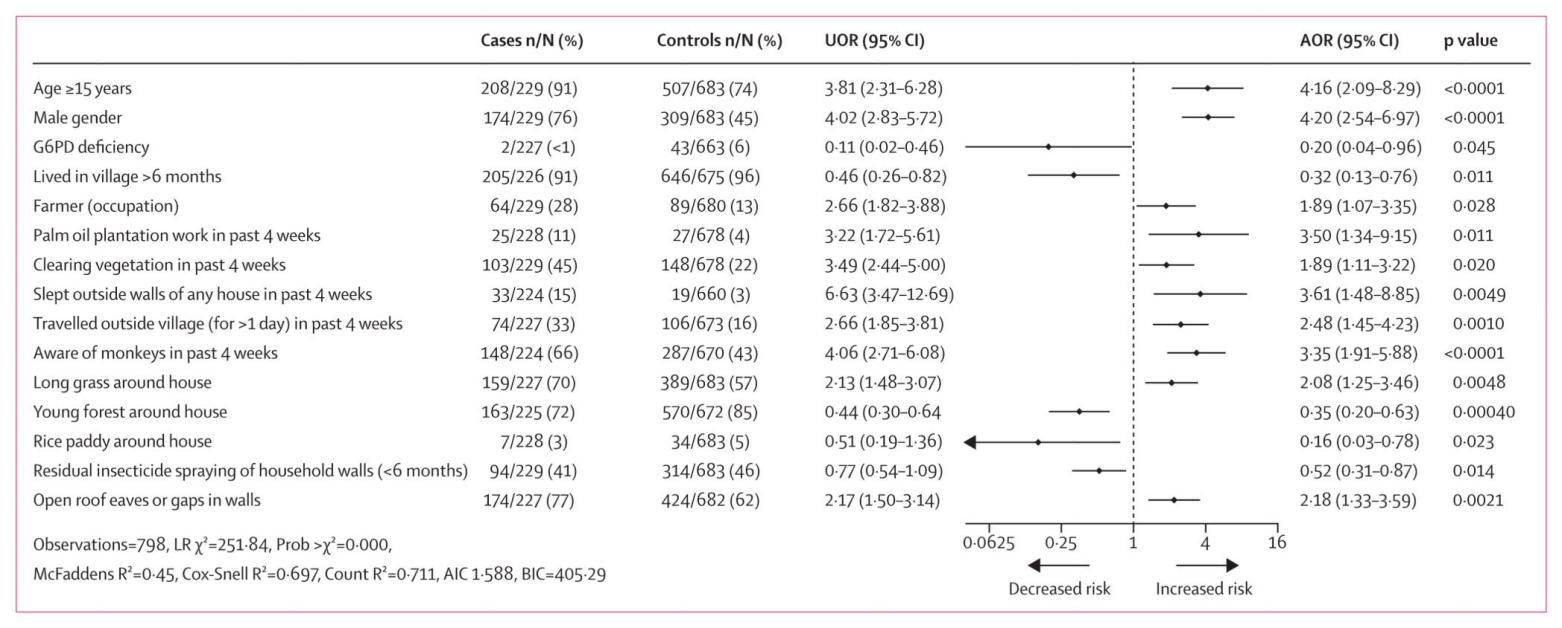
Multivariate analysis of *P knowlesi* cases and matched controls G6PD=glucose-6-phosphate dehydrogenase. UOR=unadjusted odds ratio. aOR=adjusted odds ratio. *P knowlesi*=*Plasmodium knowlesi*.

The exposure variables used for the univariate analysis and any missing data are shown in the appendix. After fitting, 15 exposure variables remained in the final multivariate model (figure 3). Adults older than 15 years were more likely to acquire knowlesi malaria compared with children (aOR 4·16, 95% CI 2·09–8·29; p<0·0001), and male gender was also associated with higher acquisition risk overall (4·20, 2·54–6·97, p<0·0001). Other intrinsic individual-level factors such as deficient G6PD enzyme activity remained protective (0·20, 0·04–0·96, p=0·045).

Identifying as a farmer was associated with an increased risk of symptomatic infection with *P knowlesi* (aOR 1·89, 95% CI 1·07–3·35, p=0·028). 47 (27%) of 174 male knowlesi cases, and 17 (31%) of 55 female knowlesi cases identified as farmers, including individuals with other primary occupations. Occupations involving work that occurs predominantly inside or around the household or other buildings, such as shopkeeping, traditional female household duties, and studying, were associated with a lower risk of malaria on univariate but not multivariate analysis. Specific occupational activities, such as palm-oil plantation work (3·50, 1·34–9·15, p=0·011) or clearing vegetation or forest in the previous month (1·89, 1·11–3·22, p=0·020) were independently associated with increased risk.

Sleeping outside was an independent acquisition risk factor (aOR 3·61, 95% CI 1·48–8·85, p=0·0049), as was a history of recent travel (2·48, 1·45–4·23, p=0·0010), although specifically sleeping outside in the forest or plantation during a trip were not significant risk factors on multivariate analysis. Residual insecticide spraying of household walls was the only malaria-prevention activity to reduce acquisition risk in the model (0·52, 0·31–0·87, p=0·014). Use of a mosquito bednet remained unassociated with protection after controlling for other variables. Interaction with monkeys, predominantly long-tailed macaques (144 [97%] of 148 cases reporting exposure to monkeys), was associated with increased acquisition risk, although higher frequency of observing monkeys did not remain significant (appendix). A history of activities taking place in the forest did not remain significantly associated with increased knowlesi malaria acquisition risk, nor did any other specific recreational activities such as hunting.

Young or sparse regenerating forest was the most commonly reported vegetation type surrounding knowlesi malaria case households (163 [72%] of 225 households) and was associated with decreased malaria risk (aOR 0·35, 95% CI 0·20–0·63, p=0·00040), as was rice paddy (0·16, 0·03–0·78, p=0·023). By contrast, the presence of long grass around the area of the household was associated with increased acquisition risk (2·08, 1·25–3·46, p=0·0048). The only household construction factor to remain in the final model was the presence of open roof eaves, large gaps, or both in the walls, which increased acquisition risk (2·18, 1·33–3·59, p=0·002), whereas bamboo walls and floors did not remain significantly associated.

**Figure 4:**
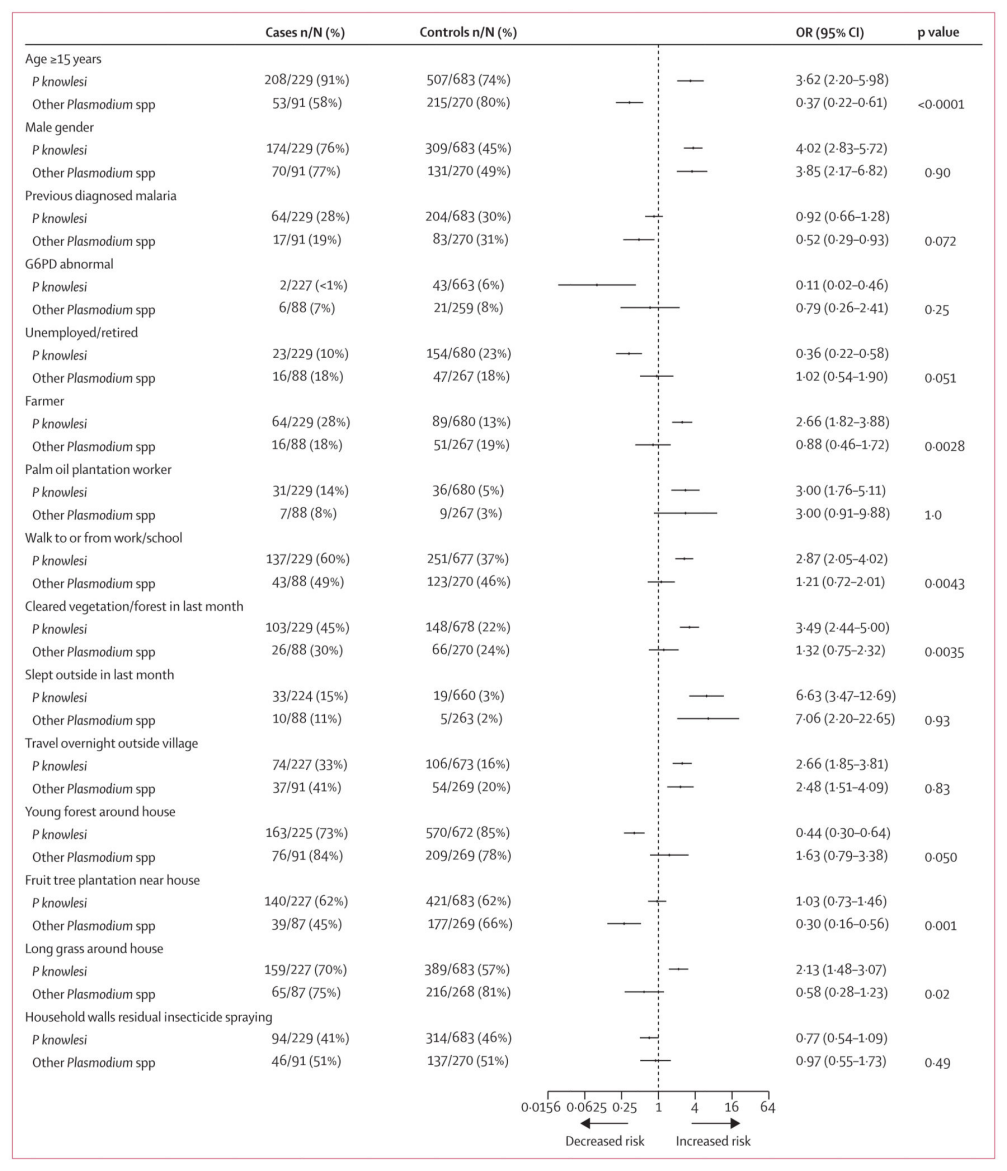
*P knowlesi* cases and matched controls versus other *Plasmodium* species cases and matched controls for selected exposure variables G6PD=glucose-6-phosphate dehydrogenase. aOR=adjusted odds ratio.

Adjusted odds ratios for exposure variables associated with non-*P knowlesi* malaria are shown in the appendix. Compared with children, adults had a lower risk of malaria from non-knowlesi *Plasmodium* spp (OR 0·37, 95% CI 0·22–0·61), but a higher risk of *P knowlesi* (3·62, 2·20–5·98, p<0·0001; figure 4). Male gender was equally associated with acquiring knowlesi malaria as non-knowlesi malaria (figure 4). Farming was a risk factor for knowlesi malaria but was not for non-knowlesi malaria (2·66, 1·82–3·88 *vs* 0·88, 0·46–1·72, respectiviely; p=0·003). Walking to work or school was also associated with increased risk of knowlesi malaria acquisition compared with non-knowlesi malaria. A history of palm-oil plantation work in the preceding 3 weeks gave a similarly increased risk of both types of malaria. Clearing vegetation or forest in the past month (3·49, 2·44–4·98) was associated with an increase in knowlesi malaria risk (1·32, 0·75–2·32; p=0·004) but not non-knowlesi malaria. Sleeping outside posed a similarly high malaria risk for knowlesi and non-knowlesi malaria, as did a history of travel. Differences existed between the malaria types in terms of type of vegetation in the immediate vicinity of the house, with young sparse forest not having a protective benefit for non-*P knowlesi* cases (1·63, 0·79–3·38), as seen in the *P knowlesi* group (0·43, 0·30–0·64; p=0·050). Having a fruit tree plantation near the house was associated with decreased risk of non-knowlesi malaria (0·30, 0·16–0·56), but not knowlesi malaria (1·03, 0·73–1·46; p=0·0071). Malaria prevention activities inside the household did not seem to be protective for non-*P knowlesi* cases, including residual insecticide spraying or mosquito bednet use.

## Discussion

In this first, comprehensive assessment of the factors associated with acquiring symptomatic *P knowlesi* infection, our key findings support the most common risk profile for knowlesi malaria, as consisting of adult men working as traditional subsistence farmers, especially those with a history of recent palm-oil plantation work. The highest individual transmission risk was at the forest-edge, including in people with a history of clearing vegetation. This finding suggests that recent ecological changes affecting the human–vegetation interface are associated with spatially heterogeneous *P knowlesi* infection,^13^,^14^ rather than exclusively interior forest exposure with activities such as hunting. Both women and children were also identified as *P knowlesi* cases, and although agricultural or other activities away from the household gave the greatest risk in these groups, we also identified peri-domestic transmission risk in the immediate vicinity of the household. Strong associations with overnight travel and sleeping outdoors emphasise the importance of population movement on zoonotic knowlesi malaria transmission. Walking to and from work also seemed to increase risk compared with human-only *Plasmodium* species transmission. Overall, these findings support previously reported data from neighbouring Sarawak^6^ and in Sabah^12^ showing that outdoor farming activities increase the risk of infection. These are areas more likely to be inhabited by incriminated mosquito vectors, including *Anopheles balabacensis*,^20^ with the ability to transmit *P knowlesi* between proximal canopy-dwelling macaques and ground-dwelling humans.^21^

Individual intrinsic factors seemed to provide protection against knowlesi malaria. In particular, phenotypic G6PD enzyme deficiency was associated with decreased risk of symptomatic infection, as seen for human-only *Plasmodium* species,^22^ and is probably related to long-term selection pressure from these human-only species. This protection is thought to be related to increased sensitivity of infected erythrocytes to parasite-induced oxidative stress, resulting in impaired parasite growth and early phagocytosis.^23^ G6PD deficiency has not been reported to occur in *M fascicularis,*^24^ and is likely to be one of several human-only host genetic factors, which, in addition to parasite factors such as normocyte binding proteins, affect human susceptibility to infection and disease severity with *P knowlesi.*^25^ The protective benefit of living in the same village for more than 6 months could be consistent with the development of immunity after more frequent exposure. However, given the low transmission levels of all *Plasmodium* species in the area,^9^ this factor might more plausibly be due to higher risk activities among recent arrivals, including clearing vegetation or constructing new houses from forest materials.

Transmission of *P knowlesi* also occurred in or around the household, as evidenced by a small number of cases, including young children, with no history of work or travel away from the house. Additionally, household factors associated with increased exposure to mosquitoes such as open eaves and gaps (particularly with bamboo floors and walls), the protective benefit of residual insecticide spraying on household walls, and a positive association between risk of infection and presence of monkeys in the vicinity of house and garden all suggest some degree of peri-domestic transmission. This notion is consistent with entomological findings from a subset of participants in this study, with *A balabacensis* being found both inside households, and more commonly (five times more) outdoors in peri-domestic areas, as well as being more abundant around case households than control households.^26^ These factors are still consistent primarily with zoonotic transmission. Although the possibility of adaptive human-to-human transmission cannot be excluded, molecular comparisons of *P knowlesi* between human and macaque hosts in other areas of Malaysia show no evidence of such transmission.^27^ Mosquito bednet use did not seem to be protective against *P knowlesi* acquisition, consistent with findings from the same study area describing earlier peak biting times of *A balabacensis* in the early evening from 1800 h to 2000 h as mosquitoes adapt to human bednet use later in the night, with biting mainly occurring outdoors.^20^ However, given that lower levels of mosquito biting have been reported to continue throughout the night,^20^ the potential of indoor transmission remains. Consequently the use of conventional prevention activities remains relevant, especially for the large proportion of people who did not use a bednet during travel away from home.

Evolutionary understanding of emerging vector-borne diseases highlights the role of land use and deforestation, with resultant changes in ecological habitats affecting vector bionomics that favour increased blood feeding on humans.^14^ Findings from another study in Sabah showed marked spatial heterogeneity of *P knowlesi* village-level case incidence that was associated with proximate forest cover and historical forest loss.^13^ Although *P knowlesi* vector sporozoite rates and parity are highest in areas of Sabah with intact forest, increased vector abundance has been reported in villages^20^ and areas of disturbed forest.^21^ In our study, occupational risk factors for infection with *P knowlesi* such as farming, palm-oil plantation work, and clearing vegetation predominantly required a short journey from the household or village. This was in contrast with human-only *Plasmodium* spp, which was not associated with these risk factors, despite both zoonotic and human-only malaria species being transmitted by the same vector in this area.^20^ Additionally, the presence of long grass and fruit trees around the household were associated with comparatively higher malaria risk in the *P knowlesi* group than in the human-only *Plasmodium* species cases, which was potentially related to closer proximity to macaques through their foraging behaviour. However, evidence suggests that the density of long-tailed macaques in Sabah is higher in areas of intact forest than in fragmented areas of oil-palm, rubber, acacia, or coconut plantations.^28^

Limitations of the study include recall bias, given that knowlesi cases who were aware of their diagnosis were potentially more likely to recall seeing monkeys or describe childhood malaria episodes. This study only enrolled symptomatic malaria cases presenting to the catchment area hospitals, and it is likely that there were people with symptomatic but spontaneously resolving *P knowlesi* infections who did not seek the free government provided treatment. However, the wide age range and geographical distribution of the cases suggests that this potential bias did not have a major effect. It has also been shown that asymptomatic submicroscopic *P knowlesi* infections can occur,^29^ with this group not seeking treatment and hence not being included in this study; however despite being unlikely, this was not excluded in controls. Stepwise selection of variables could have excluded potential weaker associations with *P knowlesi* acquisition.

Despite the variation in patterns of *P knowlesi* infection, the results of this study would seem to be generalisable to other rural forested and agricultural areas within this region with a similar profile of cultural activities, large-scale plantation or forestry-related work, subsistence farming, and recent land use change; consistent with descriptions of *P knowlesi* infection in Sumatra, Indonesia.^7^,^30^ However, extrapolation of these findings more broadly requires further data on population-level *P knowlesi* infection prevalence, complexity of land cover change, and the densities and bionomics of both primate hosts and different anopheline vectors at fine spatial scales to reliably estimate risk of potential spread or establishment of transmission in human hosts.^3^ The effects of forest fragmentation on macaque movement patterns and subsequent vector interaction and human or monkey host biting preferences remain poorly defined, but are likely to be affected by human factors assessed in this study.

Risk of knowlesi malaria is associated with a range of human interactions in farm, forest, and village environments where macaque hosts and mosquito vectors are present. Adult men working in agricultural areas had the highest risk of symptomatic *P knowlesi* infection, although peri-domestic transmission also occurred. Individual-level factors affecting zoonotic *P knowlesi* transmission in established endemic areas are potential targets in future public health interventions, which can be designed to reflect the realities of population expansion, wildlife sustainability, and agricultural development, along with ongoing promotion of conventional malaria prevention activities.

## Supplementary Material

Supplemental Data

## Figures and Tables

**Table:** Baseline demographics and features of cases and controls

	*P knowlesi* cases (n=229)	Non-*P knowlesi* cases (n=91)	*P knowlesi* matched controls (n=683)	Non-*P knowlesi* matched controls (n=270)

**Gender**				
Male	174 (76%)	70 (77%)	309 (45%)	131 (49%)
Female	55 (24%)	21 (23%)	374 (55%)	139 (51%)
**Age (years)**				
Median (IQR)	34 (24–43)	19 (11–30)	28 (14–49)	29 (17–48)
Range	3–85	2–67	1–92	1–78
Children <15 years	21 (9%)	38 (42%)	176 (26%)	55 (20%)
**Study district**				
Kudat	148 (65%)	16 (18%)	437 (64%)	48 (18%)
Kota Marudu	81 (35%)	75 (82%)	246 (36%)	222 (82%)

Data are n (%) unless indicated otherwise.

## Research in context

**Evidence before this study**

We searched PubMed between Jan 1, 2012, and March 1, 2017, with the term “knowlesi”, and then combined with “epidemiology”, “risk”, “risk-factors”, “acquisition”, case-control”, “cohort”, or “survey”, with no date restrictions for published English-language reports. We reviewed all studies identified as being related to the epidemiology of knowlesi malaria.

We identified no previous case-control studies involving human infection of *Plasmodium knowlesi*.

**Added value of this study**

To our knowledge, this is the first case-control study to assess factors associated with acquiring knowlesi malaria. Farming as a primary occupation, a history of clearing vegetation or plantation work, older age, male gender, sleeping outside, and travel history were all independently associated with increased risk of knowlesi malaria. Glucose-6-phosphate dehydrogenase deficiency and residual insecticide spraying of household walls were associated with decreased knowlesi malaria risk.

**Implications of all the available evidence**

The risk of symptomatic *P knowlesi* infection is associated with a range of human interactions in farm, forest, and village environments where macaque hosts and mosquito vectors are present. Adult men working in agricultural areas had the highest risk of *P knowlesi* infection, although peri-domestic transmission did occur, with knowlesi malaria cases including infants and people with no travel or work history.

Individual-level factors affecting zoonotic *P knowlesi* transmission in established endemic areas are potential targets for future public health interventions, along with ongoing promotion of conventional malaria prevention activities.
