# Supplementary material for: Individual-level factors associated with the risk of acquiring human Plasmodium knowlesi malaria in Malaysia: a case-control study
Source: Lancet Planet Health. Author manuscript; Available in PMC 2017 Jul 27. (PMC5531251; doi:10.1016/S2542-5196(17)30031-1)
Supplement: Supplemental Data [file NIHMS73437-supplement-Supplemental_Data.pdf]

THE LANCET
Planetary Health

Supplementary appendix

This appendix formed part of the original submission and has been peer reviewed. We post it as supplied by the authors.

Supplement to: Grigg MJ, Cox J, William T, et al. Individual-level factors associated with the risk of acquiring human *Plasmodium knowlesi* malaria in Malaysia: a case-control study. *Lancet Planetary Health* 2017; **1:** e97–104.

Table of contents:

1. **Supplementary Table 1:** Unadjusted odds ratios for all exposure variables on univariate analysis
    - 1.1 Demographics……………………………………Page 1
    - 1.2 Medical / malaria history……………………………2
    - 1.3 Work / school……………………………………………3
    - 1.4 Activities………………………………………………5
    - 1.5 Sleeping / bathing………………………………………6
    - 1.6 Malaria prevention……………………………………..7
    - 1.7 Travel history……………………………………………8
    - 1.8 Monkey interaction……………………………………..9
    - 1.9 Residential environment……………………………11
    - 1.10 Household survey…………………………………...13

2. **Supplementary Table 2:** Unadjusted and adjusted odds ratios for exposure variables associated with acquisition risk of other *Plasmodium* spp. malaria (non-*P. knowlesi*) versus matched controls………………………………………………………..14

Supplementary Table 1: Unadjusted odds ratios for all exposure variables

		P. knowlesi												Other *Plasmodium* species combined					
Variable		Case N pos	Case %	Control N pos	Control %	OR	95CI Min	95CI Max	P-val OR	Cases N tot	Controls N tot	P-val Model	r2 Model	OR	95CI Min	95CI Max	P-val OR	P-val Pk vs. non-Pk	Missing %
**Demographics**																			
Age groups																			0
	>50	62	27.1	160	23.4					229	683	0.000	0.052						
	<15	21	9.2	176	25.8	0.29	0.17	0.51	**0.000**					6.19	2.43	15.72	**0.000**	**0.045**	
	15-50	146	63.8	347	50.8	1.07	0.76	1.52	0.696					2.85	1.12	7.25	**0.028**	0.194	
Gender																			0
	Male	174	76.0	309	45.2	4.02	2.83	5.72	**0.000**	229	683	0.000	0.110	3.85	2.17	6.82	**0.000**	0.895	
Education level																			1.1
	None	67	29.9	228	33.7					224	677	0.602	0.003						
	Primary	81	36.2	232	34.3	1.23	0.82	1.74	0.343					1.25	0.73	2.15	0.412	0.952	
	Secondary	69	30.8	189	27.9	1.24	0.83	1.77	0.324					0.72	0.33	1.56	0.404	0.162	
	Tertiary	7	3.1	28	4.1	0.83	0.34	1.95	0.650					2.91	0.69	12.28	0.145	0.337	
Socio-economic status quartiles																			1.6
	Lowest 1	49	21.9	118	17.5					224	676	0.089	0.011						
	2	54	24.1	171	25.3	0.70	0.43	1.14	0.146					1.30	0.71	2.37	0.403	0.169	
	3	65	29.0	190	28.1	0.73	0.44	1.22	0.230					0.34	0.12	0.97	**0.044**	0.136	
	Highest 4	56	25.0	197	29.1	0.59	0.34	1.03	0.063					0.26	0.09	0.77	**0.016**	0.133	
Time lived in village																			0.9
	<6months	21	9.3	29	4.3					226	675	0.028	0.012						

Variable	Case N pos	Case %	Control N pos	Control %	OR	95CI Min	95CI Max	P-val OR	Cases N tot	Controls N tot	P-val Model	r2 Model	OR	95CI Min	95CI Max	P-val OR	P-val Pk vs. non-Pk	Missing %
**Medical / malaria history**																		
Previous diagnosed malaria	64	27.9	204	29.9	0.92	0.66	1.28	0.604	229	683	0.603	0.000	0.52	0.29	0.93	**0.029**	0.072	0
Took antimalarials for prevention in last 4 weeks	2	0.9	8	1.2	0.85	0.17	4.31	0.842	226	674	0.840	0.000	-					0.8
Pregnant	1	0.4	10	1.5	0.30	0.04	2.34	0.251	229	679	0.177	0.003	1.43	0.36	5.74	0.613	0.287	0.6
G6PD abnormal	2	0.2	43	4.8	0.11	0.02	0.46	**0.003**	227	663	0.000	0.027	0.79	0.26	2.41	0.681	0.250	3.2

Variable	Case N pos	Case %	Control N pos	Control %	OR	95CI Min	95CI Max	P-val OR	Cases N tot	Controls N tot	P-val Model	r2 Model	OR	95CI Min	95CI Max	P-val OR	P-val Pk vs. non-Pk	Missing %
**Work/school**																		
Occupation																		
None/unemployed/retired	23	10.0	154	22.6	0.36	0.22	0.58	**0.000**	229	680	0.000	0.033	1.02	0.54	1.90	0.957	**0.051**	0.7
Construction/contractor	8	3.5	5	0.7	4.80	1.57	14.67	**0.006**	229	680	0.005	0.012	2.33	0.66	8.28	**0.190**	0.430	0.7
Farmer	64	27.9	89	13.1	2.66	1.82	3.88	**0.000**	229	680	0.000	0.040	0.88	0.46	1.72	0.718	**0.003**	0.7
Fisherman	4	1.7	6	0.9	1.92	0.54	6.81	0.314	229	680	0.328	0.002	0.31	0.11	0.65	0.333	0.197	0.7
Housewife	11	4.8	134	19.7	0.21	0.11	0.39	**0.000**	229	680	0.000	0.055	0.26	0.03	3.31	**0.004**	0.879	0.7
Palm oil plantation worker	31	13.5	36	5.3	3.00	1.76	5.11	**0.000**	229	680	0.000	0.025	3.00	0.91	9.88	0.071	1.000	0.7
Rubber tapper	87	38.0	143	21.0	2.74	1.90	3.96	**0.000**	229	680	0.000	0.047	1.44	0.76	2.71	0.264	0.084	0.6
Shopkeeper/business	3	1.3	14	2.1	0.63	0.18	2.25	0.479	229	680	0.459	0.001	-	-	-	-	-	0.7
Student	22	9.6	129	19.0	0.45	0.28	0.73	**0.001**	229	680	0.001	0.019	1.81	0.99	3.29	0.053	**0.016**	0.7
Farmer (type)																		
Fruit	14	6.1	16	2.4	2.80	1.30	6.02	**0.008**	228	680	0.009	0.011	1.10	0.33	3.66	0.876	0.186	0.8
Coconuts	8	3.5	8	1.2	3.00	1.13	7.99	**0.028**	228	680	0.032	0.007	-	1.00	1.00			0.8
Rice	7	3.1	15	2.2	1.45	0.56	3.77	0.441	228	680	0.449	0.001	2.10	0.66	6.69	0.208	0.650	0.8
Cattle	1	0.4	2	0.3	1.50	0.14	16.54	0.741	228	680	0.747	0.000	-					0.8
Chickens	0	0.0	7	1.0	-	0.00	.	1.000	228	680	0.045	0.006	0.00	0.00	.	1.000		0.8
Swidden (slash/burn)	1	0.4	1	0.1	3.00	0.19	47.96	0.437	228	680	0.448	0.001	2.71	0.38	19.40	0.320	0.954	0.8
Gardening/vegetables	36	15.7	91	13.4	2.48	1.42	4.31	**0.001**	224	676	0.002	0.016	0.62	0.25	1.54	0.306	**0.014**	1.4
Job seasonal	36	15.7	91	13.4	1.27	0.80	2.02	0.313	229	680	0.316	0.002	1.36	0.66	2.82		0.881	0.8
Work/school location away from household	100	43.7	431	63.4	0.41	0.30	0.57	**0.000**	229	680	0.000	0.047	1.28	0.76	2.14	0.350	**0.028**	0.5
When go or return to work/school																		
Night / early morning (1am-6am)	67	29.6	150	22.1	1.52	1.08	2.15	**0.017**	226	678	0.017	0.009	1.10	0.61	1.98	0.762	0.314	0.9
Morning (7am-12pm)	148	65.5	285	42.0	2.75	1.98	3.82	**0.000**	226	678	0.000	0.063	1.41	0.84	2.37	0.192	**0.024**	0.9
Afternoon (1pm-6pm)	112	49.6	198	29.2	2.56	1.85	3.54	**0.000**	226	678	0.000	0.053	0.76	0.44	1.32	0.333	**0.000**	0.9
Evening/night (7pm-12pm)	16	7.1	16	2.4	3.29	1.57	6.90	**0.002**	226	678	0.002	0.016	1.00	0.30	3.35	1.000	0.099	0.9
Pass any type of forest/plantation on travel to work/school	220	96.1	633	92.7	2.01	0.95	4.24	**0.067**	229	683	0.052	0.006	1.14	0.50	2.57	0.761	0.355	0
Walk to or from work/school	137	59.8	251	37.1	2.87	2.05	4.02	**0.000**	229	677	0.000	0.064	1.21	0.72	2.01	0.475	**0.004**	0.7

Time to travel to or from work/school																		
<30 minutes	162	72.0	336	49.8					225	675	0.000	0.121						1.5
>30 minutes	28	12.4	40	5.9	1.85	1.03	3.29	**0.038**					1.13	0.44	2.88	0.797	0.351	
Don't travel	35	15.6	299	44.3	0.22	0.14	0.33	**0.000**					0.75	0.43	1.28	0.289	**0.013**	
Farm/plantation work type in last 4 weeks																		
Palm oil plantation worker	24	10.5	25	3.7	3.22	1.72	5.61	**0.000**	229	680	0.000	0.023	3.21	0.85	12.18	0.086	0.999	1.1
Rubber tapper	60	26.2	101	14.9	2.70	1.83	3.98	**0.000**	229	680	0.000	0.027	1.48	0.75	2.92	0.264	0.254	1.1
Fruit	5	2.2	17	2.5	0.83	0.29	2.36	0.733	228	678	0.730	0.000	-	-				1.1
Coconuts	5	2.2	8	1.2	1.88	0.61	5.73	0.270	228	678	0.285	0.002	-	-				1.1
Vegetables	7	3.1	20	2.9	1.03	0.43	2.50	0.940	228	678	0.940	0.000	0.47	0.05	4.19	0.500	0.423	1.1
Rice	8	3.5	36	5.3	0.65	0.30	1.41	0.276	228	678	0.257	0.002	0.75	0.08	6.71	0.797	0.908	1.1
Other	7	3.1	7	1.0	3.25	1.08	9.75	**0.036**	228	678	0.039	0.007	0.75	0.08	6.71	0.797	0.213	1.1
Duration of plantation work in last month																		2.4
<2 weeks	68	30.2	129	19.3					225	667	0.000	0.066						
>2 weeks	51	22.7	74	11.1	1.26	0.76	2.09	0.368					1.03	0.32	3.32	0.964	0.753	
None	106	47.1	464	69.6	0.38	0.26	0.58	**0.000**					0.79	0.41	1.50	0.471	0.064	
Cleared vegetation/forest in last month	103	45.0	148	21.8	3.49	2.44	5.00	**0.000**	229	678	0.000	0.079	1.32	0.75	2.32	0.329	**0.004**	0.6

Variable	Case N pos	Case %	Control N pos	Control %	OR	95CI Min	95CI Max	P-val OR	Cases N tot	Controls N tot	P-val Model	r2 Model	OR	95CI Min	95CI Max	P-val OR	P-val Pk vs. non-Pk	Missing %
**Activities**																		
Activities																		
Fishing	46	20.2	108	16.1	1.34	0.89	2.02	0.157	228	671	0.161	0.003	1.56	0.73	3.34	0.253	0.745	1.7
Hunting	16	7.0	9	1.3	6.26	2.56	15.30	**0.000**	228	671	0.000	0.029	3.04	1.16	7.93	**0.023**	0.317	1.7
Sport	44	19.3	100	14.9	1.39	0.93	2.07	0.107	228	671	0.112	0.004	3.12	1.70	5.73	**0.000**	0.087	1.7
Other	16	7.0	31	4.6	1.60	0.84	3.03	0.153	228	671	0.162	0.003	0.60	0.07	5.14	0.641	0.236	1.7
Frequency of primary activity																		
1-7 days	51	24.8	95	15.5					205	614	0.003	0.029						9.4
8-14 days	10	4.9	21	3.4	0.87	0.37	2.06	0.750					1.81	0.58	5.61	0.305	0.398	
15-21 days	4	1.9	12	2.0	0.70	0.18	2.70	0.608					0.46	0.04	4.82	0.518	0.741	
22-28 days	19	9.2	39	6.4	0.81	0.47	1.80	0.812					1.45	0.38	5.59	0.589	0.542	
None	122	59.2	446	72.8	0.00	0.48	1.19	**0.000**					0.42	0.21	0.84	**0.013**	0.065	
Time last did primary activity																		
<1 week ago	40	19.8	119	19.5					202	611	0.000	0.061						9.7
2 weeks ago	25	12.4	24	3.9	3.96	1.83	8.59	**0.000**					3.84	1.14	14.80	**0.083**	0.970	
3 weeks ago	8	4.0	7	1.1	5.33	1.49	19.13	**0.010**					0.95	0.84	17.65	0.952	0.339	
4 weeks ago	7	3.5	15	2.5	1.30	0.48	3.54	0.601					0.53	0.16	5.52	**0.065**	0.475	
None	122	60.4	446	73.0	0.76	0.48	1.19	0.234					4.11	0.27	1.04	**0.031**	**0.000**	
Where primary activity occurs																		
Near house	30	14.3	76	12.3	1.29	0.80	2.08	0.288	210	619	0.293	0.002	2.63	1.38	5.02	**0.003**	0.146	8.3
Near ocean	18	8.6	34	5.5	1.88	0.98	3.59	0.057	210	619	0.062	0.006	1.09	0.26	4.53	0.902	0.437	8.4
Near river	14	6.7	47	7.6	0.86	0.44	1.68	0.658	210	617	0.655	0.000	1.43	0.56	3.68	0.454	0.444	8.5
In forest	18	8.6	19	3.1	3.08	1.54	6.16	**0.002**	210	618	0.002	0.018	3.98	1.39	11.38	**0.010**	0.706	8.4
In plantation	9	4.4	12	2.0	2.40	0.97	5.94	0.059	206	609	0.065	0.006	2.15	0.40	11.44	0.369	0.909	9.4
What time usually do primary activity																		9.3
Morning (6-10am)	16	7.8	38	6.2					205	611	0.000	0.043						
Midday (11am-3pm)	6	2.9	29	4.7	0.51	0.17	1.51	0.224					2.04	0.59	7.10	0.263	0.249	
Afternoon (4-8pm)	50	24.4	90	14.7	1.18	0.58	2.41	0.645					1.89	0.18	19.30	0.593	0.757	
Night (9pm-5am)	11	5.4	8	1.3	3.04	0.97	9.55	0.057					1.37	0.22	8.57	0.736	0.446	
None	122	60.0	446	73.0	0.56	0.29	1.08	0.083					0.62	0.18	2.08	0.438	0.881	

Variable	Case N pos	Case %	Control N pos	Control %	OR	95CI Min	95CI Max	P-val OR	Cases N tot	Controls N tot	P-val Model	r2 Model	OR	95CI Min	95CI Max	P-val OR	P-val Pk vs. non-Pk	Missing %
**Sleeping / bathing**																		
Regular bedtime																		
6-7pm	10	4.5	32	4.8					224	666	0.918	0.001						2.1
8-9pm	121	54.0	378	56.8	1.09	0.51	2.34	0.829					0.58	0.24	1.41	0.228	0.310	
10-11pm	74	33.0	200	30.0	1.21	0.55	2.67	0.632					0.35	0.13	0.98	0.045	0.098	
After 11pm	19	8.5	56	8.4	1.13	0.45	2.86	0.791					0.50	0.11	2.33	0.379	0.341	
Slept outside in last month	33	14.7	19	2.9	6.63	3.47	12.69	**0.000**	224	660	0.000	0.061	7.06	2.20	22.65	**0.001**	0.928	3.0
Number of nights slept outside																		
1-7 nights	22	9.9	13	2.0					223	658	0.000	0.062						3.3
>7 nights	10	4.5	4	0.6	1.86	0.35	5.23	0.664					0.94	0.06	14.83	0.964	0.569	
None	191	85.7	641	97.4	15.15	0.07	0.34	**0.000**					0.10	0.02	0.51	**0.005**	0.000	
Normal washing place																		
Bathroom/washhouse	101	45.7	357	53.8					221	663	0.030	0.018						2.6
River	34	15.4	59	8.9	2.36	1.37	4.07	**0.002**					1.81	0.68	4.85	0.236	0.628	
Waterpipe (outside)	52	23.5	164	24.7	1.14	0.75	1.73	0.538					0.93	0.50	1.74	0.826	0.588	
Well (outside)	29	13.1	68	10.3	1.60	0.92	2.80	0.098					2.52	1.09	5.84	**0.031**	0.432	
Other	5	2.3	15	2.3	1.43	0.44	4.62	0.547					0.00	0.00	.	0.990	0.094	

Variable	Case N pos	Case %	Control N pos	Control %	OR	95CI Min	95CI Max	P-val OR	Cases N tot	Controls N tot	P-val Model	r2 Model	OR	95CI Min	95CI Max	P-val OR	P-val Pk vs. non-Pk	Missing %
**Malaria prevention**																		
Used malaria prevention in last month																		
Mosquito coils	113	49.8	302	44.6	1.27	0.92	1.75	0.143	227	677	0.142	0.003	1.03	0.63	1.68	0.901	0.468	0.9
Mosquito repellent lotion	3	1.3	7	1.0	1.18	0.30	4.58	0.811	225	675	0.813	0.000	3.00	0.19	47.96	0.437	0.673	1.3
Smoke from cardboard/wood	51	22.7	114	16.9	1.19	0.99	2.19	0.258	225	675	0.056	0.006	0.76	0.24	2.42	0.643	0.423	1.3
Kerosene lamp	12	5.4	24	3.6	1.62	0.74	3.52	0.224	223	674	0.231	0.002	3.56	0.27	47.27	0.336	0.682	1.6
Insect spray in air	70	31.0	208	30.8	1.01	0.72	1.42	0.953	226	675	0.953	0.000	0.56	0.28	1.10	0.092	0.084	1.2
Full length clothing	35	15.5	110	16.3	0.89	0.56	1.41	0.618	226	675	0.616	0.000	0.98	0.36	2.69	0.966	0.872	1.2
Mosquito bed-net	177	78.0	542	79.5	0.91	0.62	1.35	0.639	227	682	0.640	0.000	2.75	1.12	6.77	**0.027**	0.149	0.4
Indoor residual insecticide spraying in last 6 months	94	41.0	314	46.0	0.77	0.54	1.09	0.141	229	683	0.139	0.003	0.97	0.55	1.73	0.922	0.491	9.6
Insect screens fitted to doors/windows	11	4.9	43	6.3	0.75	0.38	1.49	0.413	226	683	0.402	0.001	1.00	0.32	3.10	1.000	0.694	0.6

Variable	Case N pos	Case %	Control N pos	Control %	OR	95CI Min	95CI Max	P-val OR	Cases N tot	Controls N tot	P-val Model	r2 Model	OR	95CI Min	95CI Max	P-val OR	P-val Pk vs. non-Pk	Missing %
**Travel history**																		
Stayed overnight outside village	74	32.59912	106	15.75037	2.66	1.85	3.81	**0.000**	227	673	0.000	0.045	2.48	1.51	4.09	**0.000**	0.828	1.0
Time went on trip																		
1 week ago	35	5.3	18	8.3					217	657	0.000	0.044						3.9
2 weeks ago	25	3.8	23	10.6	1.73	0.78	3.83	0.180					0.85	0.27	2.70	0.782	0.310	
3 weeks ago	17	2.6	13	6.0	1.63	0.64	4.18	0.308					1.44	0.47	4.46	0.527	0.867	
4 weeks ago	16	2.4	11	5.1	1.30	0.49	3.47	0.596					0.17	0.02	1.54	0.116	0.097	
No trip	564	85.8	152	70.0	0.52	0.29	0.96	0.035					0.37	0.16	0.85	**0.018**	0.497	
Stayed outside during trip	19	8.7	9	1.4	9.81	3.64	26.47	**0.000**	218	660	0.000	0.045	3.60	1.56	8.33	**0.003**	0.232	3.6
Stayed outside where during trip																		
Outside in forest	12	5.5	2	0.3	31.24	4.04	241.33	**0.001**	218	657	0.000	0.041	3.00	0.87	10.36	0.082	0.387	3.6
Outside in a plantation	6	2.8	4	0.6	4.97	1.22	20.17	**0.025**	217	658	0.020	0.009	2.25	0.50	10.05	0.288	0.491	3.6
Used bednet during trip	8	4.0	19	3.0	1.28	0.55	2.99	0.570	200	634	0.576	0.001	1.27	0.48	3.37	0.633	0.990	8.5

Variable	Case N pos	Case %	Control N pos	Control %	OR	95CI Min	95CI Max	P-val OR	Cases N tot	Controls N tot	P-val Model	r2 Model	OR	95CI Min	95CI Max	P-val OR	P-val Pk vs. non-Pk	Missing %
**Monkey interaction**																		
Where aware of monkeys																		
Around house	64	27.9	158	23.2	1.42	0.95	2.12	0.088	229	681	0.090	0.005	0.95	0.43	2.09	0.894	0.327	0.3
Around garden	86	37.7	131	19.2	3.63	2.42	5.44	**0.000**	228	681	0.000	0.066	1.45	0.62	3.35	0.389	**0.025**	0.4
Around village	87	38.0	161	23.6	2.43	1.67	3.53	**0.000**	229	681	0.000	0.035	1.29	0.65	2.53	0.463	0.076	0.3
In forest	84	36.7	133	19.5	2.91	1.99	4.26	**0.000**	229	681	0.000	0.049	0.89	0.38	2.05	0.778	**0.003**	0.3
Wet or dry rice paddy	19	8.3	22	3.2	3.09	1.55	6.18	**0.001**	229	681	0.001	0.016	-	-	-	-	-	0.3
Beach	7	3.1	21	3.1	0.96	0.37	2.52	0.935	228	681	0.935	0.000	2.14	0.08	6.71	0.188	0.221	0.4
Mangroves	25	10.9	41	6.0	2.41	1.29	4.53	**0.006**	229	681	0.007	0.012	0.75	0.03	3.47	0.797	0.056	0.3
Rubber plantation	17	7.4	9	1.3	7.78	3.04	19.89	**0.000**	229	681	0.000	0.034	1.00	0.08	11.93	1.000	0.085	0.4
Palm oil plantation	8	3.5	7	1.0	3.74	1.29	10.86	**0.015**	229	681	0.016	0.009	1.73	0.10	30.76	0.708	0.538	0.4
Going to work/school	27	11.8	29	4.3	3.52	1.93	6.41	**0.000**	229	681	0.000	0.027	0.54	0.15	1.92	0.343	**0.009**	0.2
Returning from work/school	30	13.1	31	4.6	3.64	2.04	6.49	**0.000**	229	681	0.000	0.031	1.11	0.32	3.80	0.873	0.048	0.2
Hunting	8	3.5	4	0.6	7.41	1.95	28.16	**0.003**	229	681	0.002	0.016	0.69	0.08	6.17	0.737	0.188	0.2
Fishing	7	3.1	8	1.2	2.91	0.96	8.81	0.060	229	681	0.062	0.006	3.00	0.42	21.30	0.272	0.978	0.2
Type of monkeys																		
Long tailed macaque	145	63.3	276	40.6	3.75	2.46	5.36	**0.000**	229	681	0.000	0.075	1.18	0.66	2.12	0.580	**0.000**	0.2
Short tailed macaque	8	3.5	4	0.6	9.77	2.04	46.75	**0.004**	229	681	0.001	0.017	3.00	0.97	9.30	0.057	0.386	0.2
How often aware of monkeys in last month																		
Once	29	12.9	67	10.0					224	670	0.000	0.106						1.7
2 times	8	3.6	30	4.5	0.59	0.24	1.50	0.269					0.37	0.07	1.98	0.246	0.597	
3 times	20	8.9	35	5.2	1.40	0.69	2.86	0.352					0.31	0.03	3.25	0.330	0.084	
>3 times	45	20.1	77	11.5	1.50	0.82	2.75	0.193					2.17	0.69	6.86	0.186	0.619	
Everyday	46	20.5	75	11.2	2.21	1.14	4.29	**0.019**					1.31	0.35	4.89	0.683	0.437	
Not aware	76	33.9	386	57.6	0.31	0.17	0.54	**0.000**					0.88	0.37	2.11	0.776	0.152	
What time aware of monkeys																		
Early morning (1am-6am)	30	13.2	36	5.5	2.70	1.58	4.59	**0.000**	227	657	0.000	0.021	2.43	0.99	5.96	0.052	0.843	2.4
Morning (7am-12pm)	96	42.3	151	23.0	2.97	2.04	4.33	**0.000**	227	657	0.000	0.055	1.04	0.54	1.97	0.912	**0.004**	2.4
Afternoon (1pm-6pm)	99	43.6	164	25.0	2.52	1.77	3.58	**0.000**	227	657	0.000	0.044	1.58	0.82	3.03	0.171	0.176	2.4
Evening/night (7pm-12am)	23	10.1	39	5.9	1.80	1.04	3.14	**0.037**	227	657	0.041	0.007	1.16	0.40	3.39	0.782	0.432	2.4

Pet monkey																		
Current pet monkey	15	6.6	14	2.0	3.94	1.74	8.88	**0.001**	229	683	0.001	0.017	1.00	0.31	3.18	1.000	0.091	0
Previous pet monkey	24	10.6	68	10.0	1.09	0.65	1.82	0.755	227	679	0.756	0.000	1.00	0.39	2.59	1.000	0.879	0.6
Other contact with monkeys in last month																		
Hunting / trapping monkeys	5	2.2	3	0.4	5.00	1.19	20.92	**0.028**	229	681	0.025	0.008	-	1.00	1.00			0.2
Chasing them from house/garden	28	12.2	29	4.3	3.44	1.93	6.12	**0.000**	229	681	0.000	0.028	3.00	0.75	12.00	0.120	0.851	0.2
Feeding monkeys	11	4.8	16	2.3	2.00	0.94	4.38	0.071	229	681	0.080	0.005	0.71	0.18	2.79	0.623	0.169	0.2

Variable	Case N pos	Case %	Control N pos	Control %	OR	95CI Min	95CI Max	P-val OR	Cases N tot	Controls N tot	P-val Model	r2 Model	OR	95CI Min	95CI Max	P-val OR	P-val Pk vs. non-Pk	Missing %
**Residential environment**																		
Forest 2 mins from house (self-reported)																		
Young forest	163	72.4	570	84.8	0.44	0.30	0.64	**0.000**	225	672	0.000	0.028	1.63	0.79	3.38	0.186	**0.050**	1.3
Secondary forest	124	55.1	441	65.6	0.61	0.43	0.85	**0.003**	225	672	0.003	0.014	0.78	0.42	1.45	0.435	0.519	1.3
Thick forest	42	18.7	102	15.2	1.33	0.86	2.05	0.203	225	672	0.207	0.003	1.64	0.64	4.21	0.302	0.686	1.3
Fallow land	49	21.8	149	22.2	1.00	0.66	1.51	1.000	225	672	1.000	0.000	0.96	0.64	4.21	0.900	0.917	1.3
Mangroves	21	9.3	53	7.9	1.22	0.66	2.24	0.525	225	671	0.528	0.001	0.60	0.07	5.14	0.641	0.415	1.3
No forest	17	7.6	13	1.9	5.74	2.34	14.08	**0.000**	225	671	0.000	0.026	0.70	0.13	3.82	0.684	0.062	1.3
Plantation 2 mins from house (self-reported)																		
Rubber	130	57.0	410	60.0	0.84	0.57	1.23	0.372	228	683	0.373	0.001	0.52	0.25	1.07	0.077	0.210	0.1
Palm oil	88	38.6	252	36.9	1.16	0.78	1.73	0.457	228	683	0.457	0.001	2.52	1.19	5.30	**0.015**	0.170	0.1
Coconut	147	64.5	466	68.2	0.81	0.56	1.16	0.256	228	683	0.258	0.002	0.49	0.25	0.96	**0.037**	0.150	0.1
Rice paddy	7	3.1	34	5.0	0.51	0.19	1.36	0.176	228	683	0.148	0.003	3.00	0.64	13.96	0.161	0.292	0.1
Fruit	140	61.7	421	61.6	1.03	0.73	1.46	0.850	227	683	0.850	0.000	0.30	0.16	0.56	**0.000**	**0.001**	0.6
None	21	9.2	39	5.7	2.04	1.04	3.98	**0.037**	229	683	0.040	0.007	2.59	0.95	7.08	0.064	0.713	0
Livestock within 50 metres of house	154	67.8	507	74.2	0.67	0.46	0.98	**0.039**	227	683	0.039	0.007	0.76	0.34	1.67	0.492	0.790	0.5
Domestic pets within 50 metres of house	204	89.9	597	87.4	1.47	0.81	2.70	0.207	227	683	0.195	0.003	0.53	0.24	1.21	0.131	0.062	0.5
Type of vegetation surrounding house																		
Fruit trees	199	87.7	603	88.3	0.95	0.54	1.66	0.846	227	683	0.847	0.000	0.19	0.08	0.47	**0.000**	**0.009**	0.6
Long grass	159	70.0	389	57.0	1.69	1.48	3.07	**0.002**	227	683	0.000	0.027	0.58	0.28	1.23	0.157	**0.015**	0.6
Forest trees	209	92.1	621	90.9	0.74	0.63	2.59	0.249	227	683	0.486	0.001	0.29	0.12	0.70	**0.006**	0.355	0.6
Forest undergrowth	13	5.7	38	5.6	1.07	0.52	2.23	0.810	227	682	0.851	0.000	0.97	0.39	2.39	0.939	0.860	0.7
Vegetable garden	15	6.6	48	7.0	0.99	0.50	1.77	0.982	227	682	0.852	0.000	1.21	0.46	3.21	0.701	0.748	0.6
House near water source																		
Flowing stream	48	21.1	136	19.9	0.90	0.73	1.73	0.577	227	683	0.593	0.000	0.52	0.25	1.08	0.079	0.230	0.6
Stagnant water	15	6.6	61	8.9	0.83	0.37	1.27	0.450	227	682	0.217	0.002	1.20	0.52	2.78	0.663	0.497	0.7
River	67	29.5	186	27.2	1.31	0.81	1.73	0.386	227	683	0.388	0.001	1.69	0.83	3.41	0.147	0.559	0.6

Lake	2	0.9	5	0.7	1.50	0.23	6.19	0.566	227	682	0.830	0.000	3.00	0.19	47.96	0.437	0.731	0.8
Ocean	12	5.3	46	6.7	0.84	0.23	1.41	0.641	227	682	0.219	0.002	2.26	0.46	11.05	0.315	0.443	0.8
Number of houses within 50m of participants residence																		
None	36	15.9	47	6.9					226	680	0.000	0.036						0.8
1 to 3	100	44.2	378	55.6	0.31	0.18	0.52	**0.000**					0.31	0.12	0.83	**0.019**	0.973	
4 to 6	33	14.6	117	17.2	0.30	0.16	0.60	**0.000**					0.30	0.10	0.87	**0.026**	0.971	
>6	57	25.2	138	20.3	0.42	0.27	0.91	**0.001**					0.30	0.10	0.84	**0.022**	0.579	

Variable	Case N pos	Case %	Control N pos	Control %	OR	95CI Min	95CI Max	P-val OR	Cases N tot	Controls N tot	P-val Model	r2 Model	OR	95CI Min	95CI Max	P-val OR	P-val Pk vs. non-Pk	Missing %
**Household survey**																		
Roof																		
Corrugated iron	219	96.5	634	92.8	2.26	1.03	4.97	**0.042**	227	683	0.028	0.008	1.53	0.46	5.03	0.486	0.570	0.6
Walls																		
Bamboo	16	7.0	21	3.1	2.55	1.26	5.17	**0.009**	227	683	0.011	0.010	1.72	0.60	4.92	0.313	0.523	0.5
Brick	2	0.9	5	0.7	1.29	0.18	9.14	0.797	227	683	0.799	0.000	1.50	0.14	16.54	0.741	0.927	0.5
Concrete/cement	28	12.3	117	17.1	0.63	0.39	1.02	0.062	227	683	0.056	0.006	0.55	0.19	1.53	0.251	0.790	0.5
Wooden	198	87.2	585	85.7	1.22	0.72	1.89	0.345	227	683	0.519	0.001	1.35	0.62	2.93	0.451	0.829	0.5
Floor																		
Bamboo	21	9.3	31	4.5	2.54	1.33	4.86	**0.005**	227	683	0.005	0.013	0.98	0.37	2.61	0.967	0.108	0.5
Concrete/cement	36	15.9	143	20.9	0.66	0.42	1.03	0.065	227	683	0.059	0.006	0.52	0.21	1.29	0.158	0.636	0.5
Tiles	10	4.4	23	3.4	1.34	0.61	2.92	0.464	227	683	0.472	0.001	0.00	0.00	.	1.000	0.012	0.5
Wooden	191	84.1	557	81.6	1.33	0.80	1.89	0.140	227	683	0.344	0.001	1.75	0.77	3.98	0.185	0.594	0.5
Open eaves	163	71.8	394	57.7	2.02	1.43	2.86	**0.000**	227	683	0.000	0.026	3.21	1.59	6.51	**0.001**	0.247	0.5
Slats / openings in floor	115	50.7	310	45.4	1.27	0.93	1.73	0.140	227	683	0.140	0.003	0.81	0.49	1.36	0.433	0.125	0.5
Gaps (>10x10cm) in house walls	120	52.9	267	39.1	1.88	1.35	2.60	**0.000**	227	682	0.000	0.023	2.45	1.46	4.12	**0.001**	0.396	0.8
Age of house																		
< 5 years	61	26.9	207	30.3					227	683	0.162	0.006						0.5
< 1 year	24	10.6	47	6.9	1.80	0.99	3.27	0.054					1.83	0.73	4.58	0.196	0.976	
> 5 years	142	62.6	429	62.8	1.12	0.78	1.60	0.542					0.80	0.43	1.47	0.467	0.349	
Toilet location outside house	173	76.2	464	67.9	1.65	1.13	2.42	**0.009**	227	683	0.008	0.011	4.20	1.43	12.34	**0.009**	0.274	0.5
Cooking area inside house	216	95.2	665	97.4	0.54	0.25	1.15	0.109	227	683	0.120	0.004	1.51	0.52	4.42	0.448	0.252	0.5
Water tank at house	178	78.4	550	80.5	0.85	0.56	1.28	0.430	227	13	0.432	0.001	0.49	0.26	0.92	**0.027**	0.137	0.5

Supplementary Table 2. Unadjusted and adjusted odds ratios for exposure variables associated with acquisition risk of other *Plasmodium* spp. malaria (non-*P. knowlesi*) versus matched controls.

	Unadjusted			Adjusted			
**Variable**	**OR**	**95% CI min**	**95% CI max**	**OR**	**CImin**	**CImax**	**P-val**
Age ≥15	0.37	0.22	0.61	**0.39**	0.19	0.78	0.008
Male gender	3.84	2.17	6.82	**3.14**	1.55	6.38	0.001
Travelled outside village (for >1 day) in last 4 weeks	2.48	1.51	4.09	**2.96**	1.33	6.60	0.008
Slept outside on trip in last 4 weeks	3.60	1.56	8.33	**3.63**	1.00	13.16	0.050
Palm-oil plantation near house	2.52	1.19	5.30	**3.36**	1.24	9.15	0.018
Wet rice paddy near house	3.00	0.64	13.96	**28.57**	3.48	234.6	0.002
Fruit trees near house	0.19	0.08	0.47	**0.14**	0.05	0.44	0.001
Open eaves in house	3.21	1.59	6.51	**5.47**	1.94	15.38	0.001
Toilet located outside	4.20	1.43	12.34	**6.30**	1.44	27.52	0.014
